# First-principles calculation of electron-phonon coupling in doped KTaO3

**DOI:** 10.12688/openreseurope.16312.1

**Published:** 2023-10-17

**Authors:** Tobias Esswein, Nicola A. Spaldin

**Affiliations:** 1 Department of Materials, ETH Zurich, Zürich, Zurich, 8093, Switzerland

**Keywords:** KTaO3, electron-phonon coupling, polarization, spin-orbit coupling

## Abstract

**Background:** Motivated by the recent experimental discovery of strongly surface-plane-dependent superconductivity at surfaces of KTaO _3_ single crystals, we calculate the electron-phonon coupling strength, *λ*, of doped KTaO _3_ along the reciprocal-space high-symmetry directions.

**Methods:**Using the Wannier-function approach implemented in the EPW package, we calculate *λ* across the experimentally covered doping range and compare its mode-resolved distribution along the [001], [110] and [111] reciprocal-space directions.

**Results:** We find that the electron-phonon coupling is strongest in the optical modes around the Γ point, with some distribution to higher *k* values in the [001] direction. The electron-phonon coupling strength as a function of doping has a dome-like shape in all three directions and its integrated total is largest in the [001] direction and smallest in the [111] direction, in contrast to the experimentally measured trends in critical temperatures.

**Conclusions:** This disagreement points to a non-BCS character of the superconductivity. Instead, the strong localization of *λ* in the soft optical modes around Γ suggests an importance of ferroelectric soft-mode fluctuations, which is supported by our findings that the mode-resolved *λ* values are strongly enhanced in polar structures. The inclusion of spin-orbit coupling has negligible influence on our calculated mode-resolved *λ* values.

## Introduction

Perovskite-structure potassium tantalate (KTaO _3_, KTO) exhibits many interesting phenomena, resulting from its high dielectric constant ^1^, strong spin orbit coupling ^2^ and charged ionic layers ^3^. The strong spin-orbit coupling (SOC), caused mainly by the heavy tantalum ion, leads to a band splitting of up to 400 meV ^2, 4^ and possible applications in spintronic devices ^5, 6^. The high dielectric constant, associated with a quantum paraelectric state ^7^ similar to that of SrTiO _3_ (STO) ^8^, indicates proximity to ferroelectricity, which is predicted to yield a large strain-dependent Rashba spin splitting ^9, 10^. The need to compensate the alternating charged ionic layers at the surfaces is predicted to induce lattice polarization in thin films ^11^, and leads to the accumulation of compensating charges at the surfaces of bulk samples ^3^. The origin and nature of the compensating charge are still open questions, with reports of conducting two-dimensional electron gases (2DEGs) ^12, 13^, charge-density waves with strongly-localized electron polarons ^14^, and terrace-like structures of alternating termination ^15^, depending on the annealing atmosphere and temperature.

**Figure 1.**
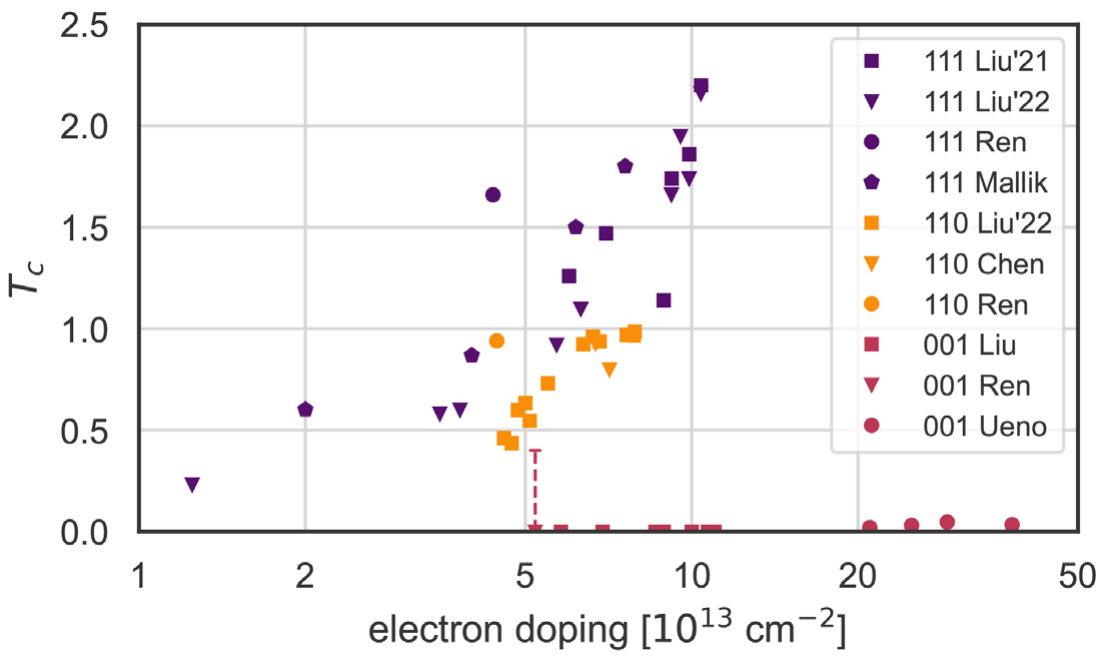
Superconducting critical temperatures, extracted from studies by Ueno *et al.* ^16^, Chen *et al.* ^19^, Liu *et al.* ^20, 39^, Ren *et al.* ^21^, and Mallik *et al.* ^40^. The (111) surface/interface reaches the highest T _c_ of up to 2 K (dark blue markers), followed by the (110) surface/interface reaching almost 1 K (bright yellow markers). The original paper by Ueno *et al.* ^16^ reported a T _c_ up to 0.05 K for the (001) surface at high doping, but more recent publications at lower doping found no (001) superconductivity down to 0.025 K ^20^ and 0.4 K ^21^ (red markers at bottom).

Perhaps the most intriguing behavior of KTO is its recently discovered low-temperature superconductivity on electron doping ^16^. Superconductivity was first achieved using ionic liquid gating on the (001) surfaces of KTO single crystals, for which critical temperatures (T _c_) of up to 50 mK were found at 2D doping concentrations of between 2 × 10 ^14^ and 4 × 10 ^14^ cm ^−2^ ^16, 17^. Note that these values correspond to 3D doping concentrations of approximately 4.1 × 10 ^20^ cm ^−3^ to 1.2 × 10 ^21^ cm ^−3^, considerably higher than the ~1.4×10 ^20^ cm ^−3^ possible using chemical doping with barium in bulk KTO ^18^. (For the conversion between 2D and 3D carrier concentrations see Ref. 16 and Figure A1 in the appendix file in the data avaliability statement). A subsequent study of LaAlO _3_-capped KTO (110) surfaces, with 2D doping concentrations of 7×10 ^13^ cm ^−2^, reached markedly higher critical temperatures up to 0.9 K ^19^; (111)-oriented KTO interfaces with either EuO or LaAlO _3_ showed even higher T _c_s of up to 2.2 K at similar carrier concentrations ^20^. Note that no superconductivity was found down to 25 mK at (001)-oriented KTO interfaces at these lower carrier concentrations ^20^. More recently, in an ionic liquid gating setup similar to that of Ref. 16, but at lower 2D doping densities of around 5 × 10 ^13^ cm ^−2^, superconductivity was found at the (110) and (111) surfaces with T _c_ of around 1 K and 2 K respectively, and not at the (001) surface down to 0.4 K ^21^. The reported critical temperatures from the literature are collected as a function of carrier concentration in Figure 1. The mechanism underlying the superconductivity, as well as its strong and unusual dependence on the orientation of the surface or interfacial plane, are not yet established. Indeed, even in the related quantum paraelectric STO, in which superconductivity was found more than half a century ago ^22, 23^, the pairing mechanism remains a subject of heated debate (for a recent review see Ref. 24). While the persistence to low carrier concentrations ^23^ and the anomalous isotope effect ^25^ challenge conventional BCS (Bardeen–Cooper–Schrieffer) theories ^26, 27^, it is likely that electron-phonon coupling in some form, as well as proximity to ferroelectricity ^28– 33^ play a role. Spin-orbit coupling has also been implicated ^34– 37^, and would be consistent with the observed higher critical temperatures in KTO, with its heavy tantalum ion, compared to STO ^32, 33, 38^. The surface-plane dependence in KTO is captured by a model in which out-of-plane polar displacements of the Ta and O ions allow a linear coupling of the transverse optical (TO) phonon to the electrons in the t _2g_ (d _xy_, d _yx_ and d _zx_) orbitals; this coupling would otherwise go to zero as the phonon wavevector *q* approached Γ ^39^. The strong dependence of the superconducting T _*c*_ on surface orientation is then explained by different inter-orbital hopping of electrons between adjacent tantalum sites via the oxygen orbitals, with the highest hopping at (111) surfaces, followed by (110) surfaces, and no hopping allowed by symmetry at (001) surfaces.

It is clear that a thorough picture of the electron-phonon coupling as a function of electron doping and throughout the Brillouin Zone in KTO is an essential step towards developing a complete theory of its superconductivity. While the electron-phonon coupling has been calculated from first principles for STO ^41^, to our knowledge it is lacking for KTO, and the goal of this work is to remedy this gap. Here we report the mode-resolved electron-phonon coupling strengths, *λ*, obtained using first-principles calculations based on density functional theory, for cubic KTaO _3_ across the range of experimentally accessible electron doping values. We extract the mode-resolved total *λ* as a function of carrier density, and focus in particular on differences between the [001], [110] and [111] high-symmetry directions, which are reciprocal to the corresponding experimentally measured surface and interfacial planes. Additionally, for one doping value, we compare the behavior with and without spin-orbit coupling, and for polar and non-polar structures to determine the effect of both properties. Our main findings are that i) the calculated total electron-phonon coupling strengths do not follow the measured trends in superconducting T _*c*_; ii) *λ* is concentrated in the optical modes around Γ and polar distortions increase *λ* by a factor of approximately five, suggesting a mechanism involving the polar soft mode; iii) spin-orbit coupling has negligible influence on the calculated electron-phonon coupling.

## Methods

To calculate the forces and total energies we use density functional theory within the generalized gradient approximation (GGA) as implemented in the Quantum ESPRESSO 7.0 and 7.1 codes ^42– 44^. We describe the exchange and correlation using the PBEsol functional ^45^, and perform the core-valence separation with the ultrasoft GBRV ^46, 47^ and pslibrary (to compare results with and without spin-orbit coupling) pseudopotentials ^48^. We use a kinetic energy cutoff of 60 Ry (816 eV) for the wave-functions, 600 Ry (8163 eV) for the charge density and a 24 × 24 × 24 k-point mesh including Γ for all unit cells. Doping is achieved in the range from 0.0001 to 0.1 electrons/formula unit (e/fu) using the background-charge method with Gaussian smearing of 1 meV width. Total energies are converged to 1 µeV (7.35 × 10 ^−8^ Ry) and forces to 0.1 meV */*Å (3.89 × 10 ^−6^ Ry */*bohr).

Both unit-cell size and shape, as well as internal coordinates, are fully relaxed, resulting in a non-polar cubic perovskite structure with a lattice constant of 3.988 Å, which is very close to the experimental one of 3.989 Å ^49^. Phonons are calculated on a 4×4×4 q-point mesh, with convergence tests on 6×6×6 and 8×8×8 q-point meshes showing only minor quantitative differences (see Figure A2 in the appendix). The resulting phonon dispersion for very low doping, using the PBEsol-relaxed unit cell, corresponds well with the room-temperature phonon dispersion calculated recently using Quantum Self-Consistent Ab Initio Lattice Dynamics (QSCAILD), which is based on DFT and a self-consistent sampling method to capture both thermal and quantum fluctuations ^50^. Note that, since our study is for doped KTO, we neglect the LO-TO splitting, assuming that it will be screened by the metallicity. To our knowledge, the evolution of the LO-TO splitting from the insulating to the metallic state as a function of doping in transition-metal oxides has not been determined, and this would be an important topic for future work.

The electron-phonon coupling properties are calculated using the EPW 5.4.1 and 5.5 codes ^51, 52^, which are included in the Quantum ESPRESSO package, and notation follows those references. The relevant electronic bands in KTaO _3_ are the three Ta-5 *d* t _2g_ bands, which are reproduced using maximally localized Wannier functions as implemented in the Wannier90 code ^53^, used internally by EPW. The electron-phonon matrix elements are first calculated on coarse 24 × 24 × 24 k-point and 4 × 4 × 4 q-point meshes and then interpolated onto fine grids using maximally localized Wannier functions. We use a random fine mesh with 1′000′000 k points to calculate the mode-resolved electron-phonon coupling strengths, *λ _qν_*, along a path between cubic high-symmetry points with 200 q points between each point. Convergence test results can be found in the appendix file in the data availability statement. Estimates of the total electron-phonon coupling strength and bulk T _*c*_ are not provided, as they would require full sampling of both k- and q-space, which we did not perform due to the excessive computational cost.

## Results and discussion

**Figure 2.**
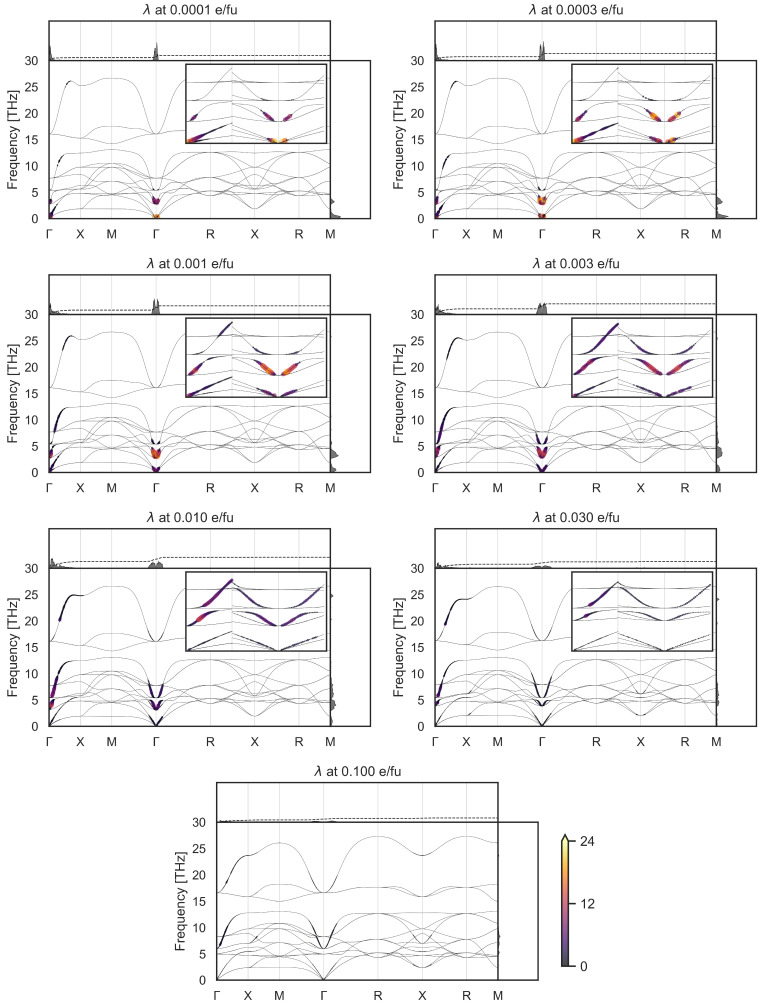
Phonon-mode-resolved electron-phonon coupling strength *λ* at different doping values ranging from 0.0001 e */*fu to 0.1 e */*fu, which correspond to 1.6 × 10 ^18^ e */*cm ^3^ to 1.6 × 10 ^21^ e */*cm ^3^ or roughly 6.2 × 10 ^12^ e */*cm ^2^ to 4.7 × 10 ^14^ e */*cm ^2^, plotted on top of phonon frequencies (narrow black lines). All plots are on the same scale and share the same colorbar for the dimensionless *λ*, shown at the bottom. The insets in the top right corners of each subplot show a zoomed-in part of the area around Γ towards the X, M and R points, ranging from from 0 THz to 10 THz. Integrated *λ* values along vertical and horizontal directions are shown in the top and right subpanels, respectively.

Our calculated mode-resolved electron-phonon coupling strengths *λ* at seven different doping levels, covering the experimental range, are shown in Figure 2, along the high symmetry directions of the Brillouin zone, with *λ* integrated at each q point shown in the top part of each subplot, and *λ* integrated over frequency (decomposed into 100 frequency steps) shown on the right of each subplot. There are several points to note. First, there are no imaginary frequencies, as the structural relaxation of KTO using the PBEsol functional results in a cubic unit cell with no structural instabilities. The frequency of the polar soft mode at Γ, which can be imaginary using the PBE functional, is 2.7 THz for the lowest doping value of 0.0001 e */*fu, and hardens to 5.0 THz at the highest doping value of 0.1 e */*fu. It has the strongest electron-phonon coupling strength *λ* throughout the whole doping range. Additionally, contributions to *λ* can be seen in the higher-energy optical modes around Γ. The strong coupling of the electrons to the polar modes at the Γ point suggests that the ferroelectric fluctuations associated with quantum paraelectricity could play a key role in the superconductivity in KTO, as already suggested for quantum paraelectric STO ^24, 28, 31^.

In the [110] (Γ to M) and [111] (Γ to R) directions, the electron-phonon coupling occurs only close to the Γ point; here the optical phonons correspond to long-wavelength ferroelectric displacements. In the [001] Γ-X direction, in contrast, the coupling, while strongest close to Γ, remains present along the entire high-symmetry line, also at higher doping. This results also in a larger total contribution along the [001] direction than along [110] and [111]. We note that the form of *λ* in reciprocal space closely follows that of the Fermi surface, which at these doping levels is close to spherical except for elongations along the cartesian reciprocal axes reflecting the flat electronic bands along Γ to X (see e.g. fig 3.3 of Ref. 54). As expected, at low doping, the electron-phonon coupling is limited largely to the lowest phonon frequencies, then extends to higher frequencies as the doping is increased and higher energy electronic bands are populated.

**Figure 3.**
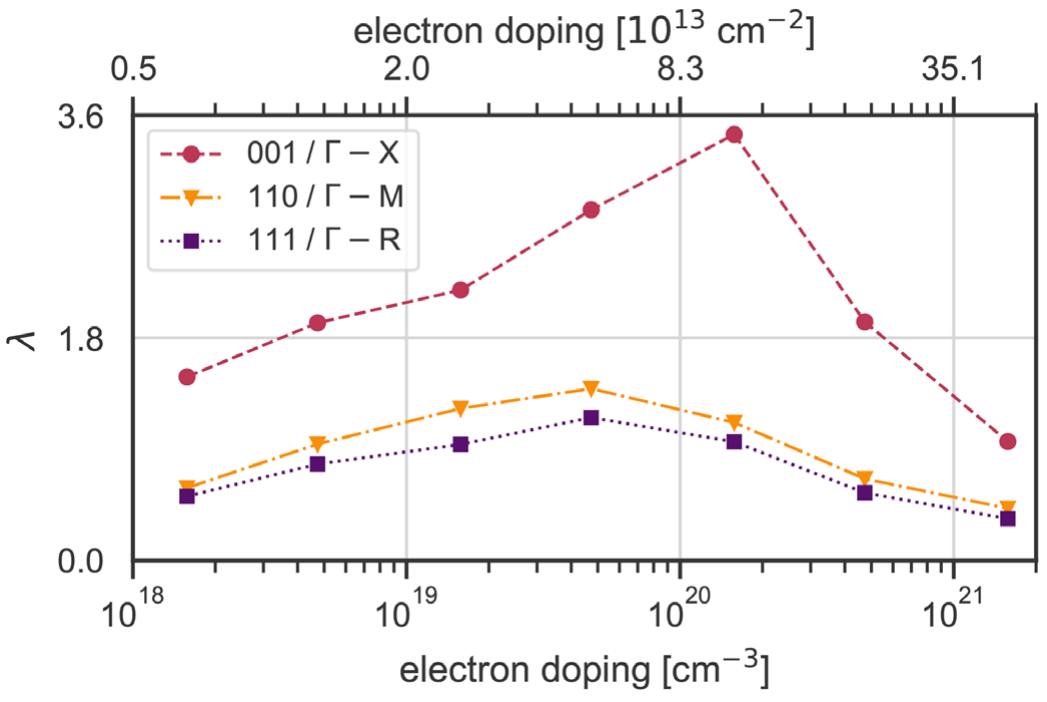
Total electron-phonon coupling strength *λ* integrated along each high-symmetry direction at different doping values, covering a range from low concentration to the maximum achieved by ionic liquid gating ^16^. The numbers on the left axis correspond to the mean lambda value of each point along each high-symmetry direction. The 2D doping values on top are estimated from the 3D values on the bottom axis using the conversion method described in section A in the appendix file. The strongest electron-phonon coupling is along the [001] direction, while the [110] and [111] directions have almost the same magnitude and evolution with doping.

The calculated integrated *λ* values along the three high-symmetry directions, which we use as proxies for the total electron-phonon coupling strength in each reciprocal direction, are shown as a function of doping concentration in Figure 3. In all directions in reciprocal space there is a dome-like structure in the calculated *λ*, with a smooth maximum between 1 × 10 ^19^ e */*cm ^−3^ to 1 × 10 ^20^ e */*cm ^−3^ (2.0 × 10 ^13^ e */*cm ^−2^ to 8.3 × 10 ^13^ e */*cm ^−2^) for the [110] and [111] directions. The more pronounced peak around 1 × 10 ^20^ e */*cm ^−3^ in the [001] direction coincides with the electron doping reaching the X point of the band structure, as can also be seen in the third row of Figure 2.

If KTO were a conventional BCS-theory superconductor, we would expect the critical temperatures of Figure 1 to follow roughly the electron-phonon coupling strength of Figure 3. Comparing those two figures, it is clear that there is no obvious correlation. First, the experimental data do not show such a dome-like trend, with the (111) surfaces/interfaces in particular showing a linear increase of T _c_ with increasing doping. Second, while experimentally the highest T _c_ is observed for the (111) surfaces/ interfaces, and the T _c_ for the (001) surfaces/interfaces is very low, the electron-phonon coupling is strongest for the [001] direction, and weakest for the [111] direction. Note that we do not consider explicitly the role of Coulomb interactions, which could also influence the anisotropy and T _*c*_s ^24^, for technical reasons, but this could be a focus of future work.

### Influence of spin-orbit coupling and polar symmetry

**Figure 4.**
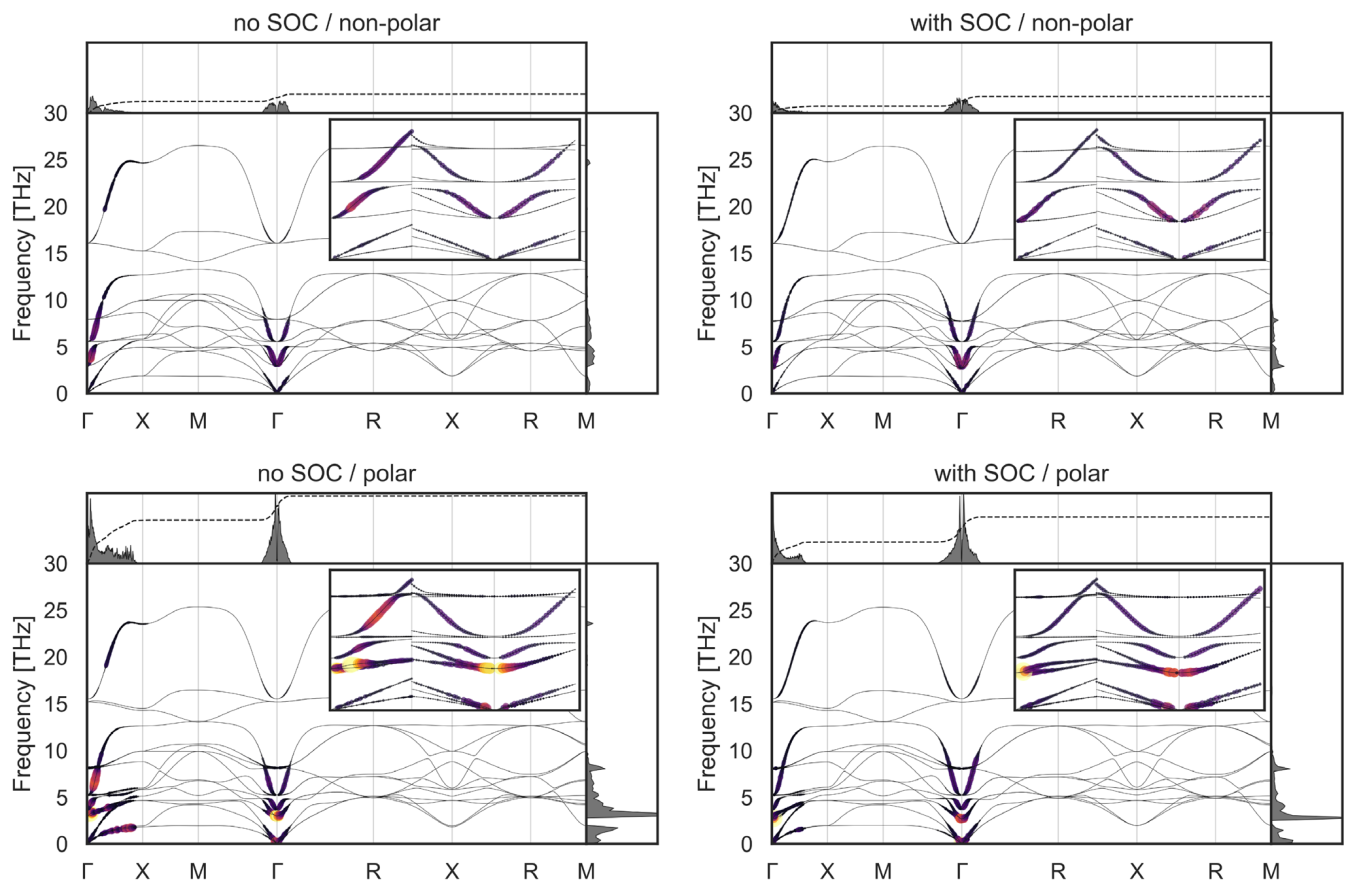
Calculated phonon-mode-resolved electron-phonon coupling strength *λ* at a doping value of 0.01 e */*fu for non-polar (top) and polar (bottom) KTO without (left) and with (right) spin-orbit coupling (SOC). All plots share the same colorbar, shown at the bottom of Figure 2, for the dimensionless *λ*.

Finally, to determine the influence of spin-orbit coupling and polar structural distortions, we present in Figure 4 our calculated mode-resolved *λ* for both non-polar and [111]-polarized KTO with and without spin-orbit coupling (SOC). (The influence of SOC on the electronic bands is shown in Figure A4 in the appendix.) The polar structure is obtained by increasing the cubic lattice constant to 4.010 Å and relaxing the internal coordinates; the soft mode frequency is then close to that of the original cubic structure (~3.0 THz).

First we compare the results with and without SOC in the cubic structures (top row) ^1^ and see that the differences are negligible. In contrast, the differences between the non-polar and polar structures (down the columns) are substantial. First, the integrated values of *λ* in the polar structures are larger by a factor of around five than the non-polar values. Second, the lowest energy branches of the soft TO modes around Γ, which had negligible electron-phonon coupling in the cubic structures, are now among the main contributors to *λ*. Third, coupling along the Γ-X direction is enhanced by the polar distortion, especially in the polar case without SOC. Note that in the polar case, spin-orbit coupling slightly reduces the *λ* values.

**Figure 5.**
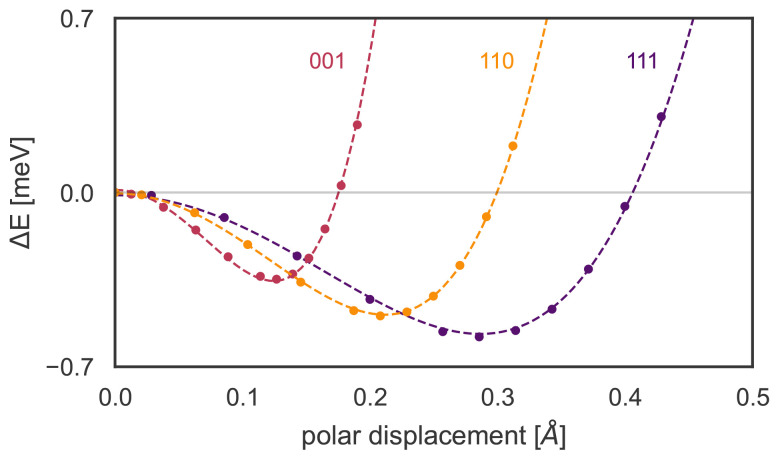
Calculated energy as a function of polar distortion along the high-symmetry directions in the doped (0.01 e */*fu) KTO unit cell, as used in the bottom left of Figure 4 (‘no SOC/polar’). The horizontal axis shows the summed displacement of all atoms in a unit cell relative to their non-polar positions. A polarization along the [111] direction has both the largest energy gain and the largest displacement.

The fact that the polar symmetry breaking has such a large calculated effect on *λ* is consistent with recent reports of polar symmetry breaking coexisting with superconductivity in STO ^55, 56^ and theories of coupling to polar modes in KTO ^57^. Note that the tendency of KTO to become polar is strongest along the [111] direction, and weakest along the [001] direction, as shown in Figure 5. This trend is consistent with that of the measured T _c_, again pointing to a possible relevance of the ferroelectric soft mode.

### Summary

In summary, we have calculated the electron-phonon coupling in KTaO _3_ for electron dopings between 1.6×10 ^18^ e */*cm ^3^ and 1.6 × 10 ^21^ e */*cm ^3^ and analyzed the results in light of the recently reported superconductivity and its surface dependence. Our calculations indicate that the measured trends in superconducting T _*c*_ are not reflected in the calculated electron-phonon coupling strengths *λ* along the corresponding reciprocal directions, confirming earlier suggestions that the superconductivity is not bulk BCS-like in nature ^7, 17, 20, 58^. In this context, recent angle-resolved photoemission spectroscopy measurements implicating coupling of bulk-like electrons to Fuchs-Kliewer *surface* phonons are highly relevant ^59^. The concentration of *λ* in the lowest frequency optical modes close to Γ hints towards a mechanism in which the polar soft mode plays a role. A comparison of the mode-resolved *λ* values between non-polar and polar structures shows clearly that polarization strongly enhances the electron-phonon coupling. In contrast, a comparison of mode-resolved *λ* values between calculations without and with spin-orbit coupling shows negligible difference. 

## Data Availability

Materials cloud. “First-principles calculation of electron-phonon coupling in doped KTaO3”. DOI: 10.24435/materialscloud:3t-k3. This project contains the following underlying data: README.txt materialscloud_KTO_EPW.zip (zip file containing all input files) Appendix.pdf (Additional computational and convergence details) Creative Commons Attribution 4.0 International license (CC-BY 4.0).

## Footnotes

^1^Note the small difference between the ‘no SOC/non-polar’ panel of Figure 4 and the corresponding ‘ *λ* at 0.010 e/fu’ panel of Figure 2. The difference arises from the fact that for Figure 4 we calculated everything using scalar-relativistic pslibrary pseudopotentials to make it directly comparable to the SOC results which we obtained using fully-relativistic pslibrary pseudopotentials.

## References

[1] Barrett JH : Dielectric Constant in Perovskite Type Crystals. *Phys Rev.* 1952;86(1):118–120. 10.1103/PhysRev.86.118

[2] Uwe H Oka K Unoki H : Raman Scattering from Conduction Electrons in KTaO3. *J Phys Soc Jpn.* 1980;49(Suppl. A):577–580.

[3] Stengel M : Electrostatic stability of insulating surfaces: Theory and applications. *Phys Rev B.* 2011;84(20): 205432. 10.1103/PhysRevB.84.205432

[4] Mattheiss LF : Energy Bands for KNiF _3_, SrTiO _3_, KMoO _3_, and KTaO _3_. *Phys Rev B.* 1972;6(12):4718–4740. 10.1103/PhysRevB.6.4718

[5] Nakamura H Kimura T : Electric field tuning of spin-orbit coupling in ktao _3_ field-effect transistors. *Phys Rev B.* 2009;80(12): 121308. 10.1103/PhysRevB.80.121308

[6] Gupta A Silotia H Kumari A : KTaO _3_—The New Kid on the Spintronics Block. *Adv Mater.* 2022;34(9): e2106481. 10.1002/adma.202106481 34961972

[7] Rowley SE Spalek LJ Smith RP : Ferroelectric quantum criticality. *Nat Phys.* 2014;10(5):367–372. Reference Source

[8] Müller KA Burkard H : SrTiO _3_: An Intrinsic Quantum Paraelectric below 4 K. *Phys Rev B.* 1979;19(7):3593–3602. 10.1103/PhysRevB.19.3593

[9] Tao LL Wang J : Strain-tunable ferroelectricity and its control of Rashba effect in KTaO _3_. *J Appl Phys.* 2016;120(23): 234101. 10.1063/1.4972198

[10] Gastiasoro MN Temperini ME Barone P : Generalized Rashba Electron-Phonon Coupling and Superconductivity in Strontium Titanate. *Phys Rev Res.* 2022;5: 023177. 10.1103/PhysRevResearch.5.023177

[11] Gattinoni C Spaldin NA : Prediction of a strong polarizing field in thin film paraelectrics. *Phys Rev Res.* 2022;4(3): L032020. 10.1103/PhysRevResearch.4.L032020

[12] King PDC He RH Eknapakul T : Subband structure of a two-dimensional electron gas formed at the polar surface of the strong spin-orbit perovskite ktao _3_. *Phys Rev Lett.* 2012;108(11): 117602. 10.1103/PhysRevLett.108.117602 22540511

[13] Santander-Syro AF Bareille C Fortuna F : Orbital symmetry re-construction and strong mass renormalization in the two-dimensional electron gas at the surface of ktao _3_. *Phys Rev B.* 2012;86(12): 121107(R). 10.1103/PhysRevB.86.121107

[14] Reticcioli M Wang Z Schmid M : Competing electronic states emerging on polar surfaces. *Nat Commun.* 2022;13(1): 4311. 10.1038/s41467-022-31953-6 35879300 PMC9314351

[15] Setvin M Reticcioli M Poelzleitner F : Polarity compensation mechanisms on the perovskite surface KTaO _3_ (001). *Science.* 2018;359(6375):572–575. 10.1126/science.aar2287 29420289

[16] Ueno K Nakamura S Shimotani H : Discovery of superconductivity in KTaO _3_ by electrostatic carrier doping. *Nat Nanotechnol.* 2011;6(7):408–412. 10.1038/nnano.2011.78 21602813

[17] Ueno K Shimotani H Yuan H : Field-Induced Superconductivity in Electric Double Layer Transistors. *J Phys Soc Jpn.* 2014;83(3): 032001. 10.7566/JPSJ.83.032001

[18] Sakai A Kanno T Yotsuhashi S : Thermoelectric Properties of Electron-Doped KTaO _3_. *Jpn J Appl Phys.* 2009;48(9R): 097002. 10.1143/JJAP.48.097002

[19] Chen Z Liu Z Sun Y : Two-dimensional superconductivity at the laalo _3_/ktao _3_(110) heterointerface its control of Rashba effect in KTaO _3_. *Phys Rev Lett.* 2021;126(2): 026802. 10.1103/PhysRevLett.126.026802 33512194

[20] Liu C Yan X Jin D : Two-dimensional superconductivity and anisotropic transport at KTaO _3_ (111) interfaces. *Science.* 2021;371(6530):716–721. 10.1126/science.aba5511 33479119

[21] Ren T Li M Sun X : Two-dimensional superconductivity at the surfaces of KTaO _3_ gated with ionic liquid. *ArXiv220100990 Cond-Mat.* 2022. Reference Source 10.1126/sciadv.abn4273 PMC9166623 35658041

[22] Schooley JF Hosler WR Cohen ML : Superconductivity in Semiconducting SrTiO _3_. *Phys Rev Lett.* 1964;12(17):474–475. 10.1103/PhysRevLett.12.474

[23] Schooley JF Hosler WR Ambler E : Dependence of the superconducting transition temperature on carrier concentration in semiconducting srtio _3_. *Phys Rev Lett.* 1965;14(9):305–307. 10.1103/PhysRevLett.14.305

[24] Gastiasoro MN Ruhman J Fernandes RM : Superconductivity in dilute SrTiO _3_: A review. *Ann Phys (N Y).* 2020;417: 168107. 10.1016/j.aop.2020.168107

[25] Stucky A Scheerer GW Ren Z : Isotope effect in superconducting n-doped srtio _3_. *Sci Rep.* 2016;6: 37582. 10.1038/srep37582 27892485 PMC5124855

[26] Bardeen J Cooper LN Schrieffer JR : Microscopic Theory of Superconductivity. *Phys Rev.* 1957;106(1):162–164. 10.1103/PhysRev.106.162

[27] Bardeen J Cooper LN Schrieffer JR : Theory of Superconductivity. *Phys Rev.* 1957;108(5):1175–1204. 10.1103/PhysRev.108.1175

[28] Edge JM Kedem Y Aschauer U : Quantum Critical Origin of the Superconducting Dome in SrTiO _3_. *Phys Rev Lett.* 2015;115(24): 247002. 10.1103/PhysRevLett.115.247002 26705650

[29] Ruhman J Lee PA : Superconductivity at very low density: The case of strontium titanate. *Phys Rev B.* 2016;94(22): 224515. 10.1103/PhysRevB.94.224515

[30] Coak MJ Haines CRS Liu C : Pressure dependence of ferroelectric quantum critical fluctuations. *Phys Rev B.* 2019;100(21): 214111. 10.1103/PhysRevB.100.214111

[31] van der Marel D Barantani F Rischau CW : Possible mechanism for superconductivity in doped SrTiO _3_. *Phys Rev Research.* 2019;1(1): 013003. 10.1103/PhysRevResearch.1.013003

[32] Gastiasoro MN Trevisan TV Fernandes M : Anisotropic superconductivity mediated by ferroelectric fluctuations in cubic systems with spin-orbit coupling. *Phys Rev B.* 2020;101(17): 174501. 10.1103/physrevb.101.174501

[33] Gastiasoro MN Temperini ME Barone P : Theory of superconductivity mediated by Rashba coupling in incipient ferroelectrics. *Phys Rev B.* 2022;105(22): 224503. 10.1103/PhysRevB.105.224503

[34] Kozii V Fu L : Odd-Parity Superconductivity in the Vicinity of Inversion Symmetry Breaking in Spin-Orbit-Coupled Systems. *Phys Rev Lett.* 2015;115(20): 207002. 10.1103/PhysRevLett.115.207002 26613464

[35] Kanasugi S Yanase Y : Spin-orbit-coupled ferroelectric superconductivity. *Phys Rev B.* 2018;98(2): 024521. 10.1103/PhysRevB.98.024521

[36] Kanasugi S Yanase Y : Multiorbital ferroelectric superconductivity in doped SrTiO _3_. *Phys Rev B.* 2019;100(9): 094504. 10.1103/PhysRevB.100.094504

[37] Kozii V Bi Z Ruhman J : Super-conductivity near a Ferroelectric Quantum Critical Point in Ultralow-Density Dirac Materials. *Phys Rev X.* 2019;9(3): 031046. 10.1103/PhysRevX.9.031046

[38] Yu Y Hwang HY Raghu S : Theory of superconductivity in doped quantum paraelectrics. *npj Quantum Mater.* 2022;7(1):63. 10.1038/s41535-022-00466-2

[39] Liu C Zhou X Hong D : Tunable superconductivity at the oxide-insulator/KTaO _3_ interface and its origin.2022. 10.48550/arXiv.2203.05867

[40] Mallik S Ménard GC Saïz G : Superfluid stiffness of a KTaO _3_-based two-dimensional electron gas. *Nat Commun.* 2022;13(1):4625. 10.1038/s41467-022-32242-y 35941153 PMC9360446

[41] Zhou JJ Hellman O Bernardi M : Electron-phonon scattering in the presence of soft modes and electron mobility in SrTiO _3_ perovskite from first principles. *Phys Rev Lett.* 2018;121(22): 226603. 10.1103/PhysRevLett.121.226603 30547621

[42] Giannozzi P Baroni S Bonini N : QUANTUM ESPRESSO: A modular and open-source software project for quantum simulations of materials. *J Phys Condens Matter.* 2009;21(39): 395502. 10.1088/0953-8984/21/39/395502 21832390

[43] Giannozzi P Andreussi O Brumme T : Advanced capabilities for materials modelling with Quantum ESPRESSO. *J Phys Condens Matter.* 2017;29(46): 465901. 10.1088/1361-648X/aa8f79 29064822

[44] Giannozzi P Baseggio O Bonfà P : Quantum ESPRESSO toward the exascale. *J Chem Phys.* 2020;152(15): 154105. 10.1063/5.0005082 32321275

[45] Perdew JP Ruzsinszky A Csonka GI : Restoring the Density-Gradient Expansion for Exchange in Solids and Surfaces. *Phys Rev Lett.* 2008;100(13): 136406. 10.1103/PhysRevLett.100.136406 18517979

[46] Garrity KF Bennett JW Rabe KM : Pseudopotentials for high-throughput DFT calculations. *Comput Mater Sci.* 2014;81:446–452. 10.1016/j.commatsci.2013.08.053

[47] Garrity KF Bennett JW Rabe KM : GBRV pseudopotentials.2019. Reference Source

[48] Dal Corso A : Pseudopotentials periodic table: From H to Pu. *Computational Materials Science.* 2014;95:337–350. 10.1016/j.commatsci.2014.07.043

[49] Wemple SH : Some transport properties of oxygen-deficient single-crystal potassium tantalate (KTaO _3_). *Phys Rev.* 1965;137(5A):A1575–A1582. 10.1103/PHYSREV.137.A1575

[50] Meier QN Mingo N van Roekeghem A : Finite temperature dielectric properties of KTaO _3_ from first principles and machine learning: Phonon spectra, barrett law, strain engineering and electrostriction.2022. 10.48550/arXiv.2206.08296

[51] Giustino F Cohen ML Louie SG : Electron-phonon interaction using Wannier functions. *Phys Rev B.* 2007;76(16): 165108. 10.1103/PhysRevB.76.165108 17358802

[52] Poncé S Margine ER Verdi C : EPW: Electron–phonon coupling, transport and superconducting properties using maximally localized Wannier functions. *Comput Phys Commun.* 2016;209:116–133. 10.1016/j.cpc.2016.07.028

[53] Pizzi G Vitale V Arita R : Wannier90 as a community code: New features and applications. *J Phys Condens Matter.* 2020;32(16): 165902. 10.1088/1361-648X/ab51ff 31658458

[54] Esswein T : Exploring Ferroelectric Quantum Criticality and Superconductivity in KTaO3. Master Thesis.2018. 10.3929/ethz-b-000560845

[55] Salmani-Rezaie S Ahadi K Stemmer S : Polar Nanodomains in a Ferroelectric Superconductor. *Nano Lett.* 2020;20(9):6542–6547. 10.1021/acs.nanolett.0c02285 32786945

[56] Salmani-Rezaie S Jeong H Russell R : Role of locally polar regions in the superconductivity of SrTiO _3_. *Phys Rev Materials.* 2021;5(10): 104801. 10.1103/PhysRevMaterials.5.104801

[57] Venditti G Temperini ME Barone P : Anisotropic Rashba coupling to polar modes in KTaO _3_ .2022. 10.48550/arXiv.2211.05056

[58] Klein A Kozii V Ruhman J : A theory of criticality for quantum ferroelectric metals.2022.

[59] Chen X Yu T Liu Y : Orientation-dependent electron-phonon coupling in interfacial superconductors LaAlO3/KTaO3.2023. 10.48550/arXiv.2301.13488

