# Supplementary material for: First-principles calculation of electron-phonon coupling in doped KTaO3
Source: Open Res Eur. 2023 Oct 17;3:177. [Version 1] doi: 10.12688/openreseurope.16312.1 (PMC10728587; doi:10.12688/openreseurope.16312.1)
Supplement: Peer review history

# Reviewer report: Reviewer response for version 1

**DOI:** 10.21956/openreseurope.17609.r36500

**Published:** 2023-12-21

**Authors:** Jon Lafuente-Bartolome

**Affiliations:** 1 University of the Basque Country (UPV/EHU), Bilbao, Spain

The authors present an ab initio analysis of electron-phonon interactions as a function of doping in the quantum paraelectric KTaO3. This material has attracted increased interest recently, as it has been found experimentally that it shows a strong surface-orientation dependent interfacial superconductivity upon doping. 

 The manuscript introduces the topic with a comprehensive and clear introduction to the recent literature in this field, and in general I found that the article is well written and accessible for a broader audience. The ab initio calculations presented here constitute an important first step towards a better theoretical understanding of superconductivity in this material, and might serve as a very useful reference for future works in this direction. 

 However, I believe that there are some technical aspects that should be clarified by the authors so that this manuscript can serve as a solid reference: 

 - The authors use ultrasoft pseudopotentials from the pslibrary and GBRV libraries. Although the results might be correct, currently only norm-conserving pseudopotentials are officially supported by EPW (see npj Comput Mater 9, 156 (2023) section “Computational workflow and basic capabilities”). I would suggest testing the presented results, at least for one doping level, against calculations using norm-conserving pseudopotentials. 

 - In Fig. 5, the authors show the energy surface as a function of polar displacements. They find a clear double-well structure, showing that distortions along any of the three directions minimize the total energy towards a polar structure. In this case, how are the frequencies of the corresponding TO modes not imaginary? I would assume that including anharmonic effects would be needed to obtain a dynamically stable structure and well-defined phonons (see e.g. Adv Quantum Technol. 6, 2200131 (2023)). 

 - From the convergence tests shown in the Appendix, it seems that the calculations are not converged with respect to the coarse-q mesh. The authors mention that “actual \lambda values are about twice as large in the q6 and q8 cases compared to the q4 case”. I believe that at least 6x6x6 coarse-q grids should be used for the calculations presented in the manuscript. 

 - The integrated \lambda values shown in Fig. 3 are quite large. They would result in relatively high Tc estimates following e.g. the McMillan-Allen-Dynes formula, and as far as I understand, no bulk superconductivity has been measured in bulk KTaO3. Could the authors comment on this? 

 - In the top and right subpanels of Figs. 2 and 4, the axes have no labels, and the shaded areas are invisible in some cases. What is the dashed line in the top subpanel? It might be more useful to define the Eliashberg function in the right panel, from which the integrated coupling strength might be obtained and visualized as a function of frequency (see e.g. Eq. (205)-(206) of Rev. Mod. Phys. 89, 015003). Moreover, I would suggest labeling the mode and momentum dependent electron-phonon interaction strength as \lambda_{q\nu} throughout the text and figures, to discern it from the total integrated coupling strength \lambda.

Is the study design appropriate and does the work have academic merit?

Yes

Is the work clearly and accurately presented and does it cite the current literature?

Yes

If applicable, is the statistical analysis and its interpretation appropriate?

Not applicable

Are all the source data underlying the results available to ensure full reproducibility?

Yes

Are the conclusions drawn adequately supported by the results?

No

Are sufficient details of methods and analysis provided to allow replication by others?

Yes

Reviewer Expertise:

Computational Condensed Matter Physics; Electron-phonon interactions; Polarons;

I confirm that I have read this submission and believe that I have an appropriate level of expertise to confirm that it is of an acceptable scientific standard, however I have significant reservations, as outlined above.

# Reviewer report: Reviewer response for version 1

**DOI:** 10.21956/openreseurope.17609.r36498

**Published:** 2023-12-18

**Authors:** Zhen Tong

**Affiliations:** 1 Sun Yat-Sen University, Shenzhen, China

In this manuscript, the authors studied the electron-phonon coupling strength of doped KTaO _3_ along the reciprocal space high symmetry directions. They discovered that in contrast to the experimentally measured trends in critical temperatures, the electron-phonon coupling strength as a function of doping has a dome-like shape in all three directions, which points to a non-BCS character of the superconductivity. This work is very interesting and meaningful. It should address the following improvements before the publication.

 (1) It is recommended to add a schematic diagram of the KTO crystal structure to the appendix so that even readers unfamiliar with perovskites can understand it.

 (2) The authors discussed the calculated total electron-phonon coupling strengths do not follow the measured trends in superconducting T _c_ because KTO is not a conventional BCS-theory superconductor. Could the authors explain this in more detail? Which other theory do the authors think is more reasonable to explain the superconductivity of KTO?

 (3) In Fig. 4, the effect of SOC on the non-polar structure is negligible, but the effect of SOC on the polar structure is obvious. Perhaps the authors could add new curves ( *Calculated energy as a function of polar distortion along the high-symmetry directions in the doped KTO unit cell, ‘SOC/polar’*) to Figure 5 to illustrate how SOC affects the polar structure.

 (4) In the Title and Abstract, it should be “KTaO _3_”, not “KTaO3”.

Is the study design appropriate and does the work have academic merit?

Yes

Is the work clearly and accurately presented and does it cite the current literature?

Yes

If applicable, is the statistical analysis and its interpretation appropriate?

Partly

Are all the source data underlying the results available to ensure full reproducibility?

Partly

Are the conclusions drawn adequately supported by the results?

Partly

Are sufficient details of methods and analysis provided to allow replication by others?

Partly

Reviewer Expertise:

electron-phonon coupling transport

I confirm that I have read this submission and believe that I have an appropriate level of expertise to confirm that it is of an acceptable scientific standard, however I have significant reservations, as outlined above.

# Reviewer report: Reviewer response for version 1

**DOI:** 10.21956/openreseurope.17609.r36513

**Published:** 2023-12-15

**Authors:** Xuetao Zhu

**Affiliations:** 1 Institute of Physics,, Chinese Academy of Sciences, Beijing, China; 2 School of Physical Sciences, University of Chinese Academy of Sciences, Beijing, China

The superconductivity discovered in doped KTaO _3_ has attracted a lot of attention. Especially, the unique orientation-dependent *T*_C_ provides an interesting perspective to view the mechanism of superconductivity.

 The paper entitled “First-principles calculation of electron-phonon coupling in doped KTaO _3_” by Tobias Esswein and Nicola A. Spaldin, reports a detailed study by first-principles calculation of the electron-phonon coupling (EPC) strength along different directions in doped KTaO _3_. The authors discovered that the EPC in the [001] direction is stronger than that in the [110] and [111] directions, which is not consistent with the experimentally measured orientation-dependent trend of *T*_C_. Based on this contrast, the authors claim that the superconducting mechanism of doped KTaO _3_ is not a conventional BCS-like picture. In this work, the calculations are carefully performed and the study of the EPC in doped KTaO _3_ will provide useful information for the understanding of the superconducting mechanism. After carefully reading the manuscript, I am still puzzled by the following aspects. I hope the authors can give more explanations.

  

1. Since I am not an expert on first-principles calculations, I cannot evaluate the accuracy of the EPC constants obtained by the authors. However, it seems the EPC constants obtained in Figure 3, especially the ones in the [001] direction (with the highest value up to ~3.5), are much larger than commonly reported EPC constants of any material, at least according to my knowledge. If this is true, such strong EPC must have some nonnegligent effect on the properties of KTaO _3_. But I don’t notice any reports of the interesting properties related to such strong EPC in KTaO _3_.
2. It seems all the EPC calculations are performed in the framework of the Eliashberg picture, which is usually applicable for metals with the Fermi energy ( *E*_F_) much larger than the phonon energy ( *ħ* *ω*), i.e., *E*_F_>> *ħ* *ω*, in an adiabatic EPC limit. However, for the doped KTaO _3_, the *E*_F_ should be much lower than conventional metals. For example, in Ref.[39] (Nature Communications, 14, 951 (2023)), the *E*_F_ of the doped KTaO _3_ was estimated to lie in the range of 10−80 meV, in the same energy range as the phonon energies of KTaO _3_. In this case ( *E*_F_ comparable with *ħ* *ω*), the EPC should be in the nonadiabatic limit. I am not sure if the nonadiabatic effect was included in the calculations of the current work.

By the way, there are several format errors in the References:

1. Lots of chemical formulas are not capitalized. For example, Refs. [5] [13] [19] and [25].
2. The paper title in Ref. [19] is not correct.
3. The journal information of several references is missing, e.g., Refs. [39] [50] [57] [58].

Please carefully check all the references.

Is the study design appropriate and does the work have academic merit?

Yes

Is the work clearly and accurately presented and does it cite the current literature?

Yes

If applicable, is the statistical analysis and its interpretation appropriate?

Not applicable

Are all the source data underlying the results available to ensure full reproducibility?

No source data required

Are the conclusions drawn adequately supported by the results?

Yes

Are sufficient details of methods and analysis provided to allow replication by others?

Yes

Reviewer Expertise:

Surface Phonon; Plasmon; Electron-Phonon Coupling; High-Resolution Electron Energy Loss Spectroscopy; Helium Atom Scattering

I confirm that I have read this submission and believe that I have an appropriate level of expertise to confirm that it is of an acceptable scientific standard, however I have significant reservations, as outlined above.

# Reviewer report: Reviewer response for version 1

**DOI:** 10.21956/openreseurope.17609.r35727

**Published:** 2023-12-15

**Authors:** Matthias Geilhufe

**Affiliations:** 1 Chalmers University of Technology, Chalmers, Sweden

"The manuscript titled 'First-Principles Calculation of Electron-Phonon Coupling in Doped KTaO3' authored by Esswein and Spaldin presents state-of-the-art ab initio calculations regarding the strength of electron-phonon coupling in KTaO _3 _(KTO). In its pristine state, KTO behaves as a quantum paraelectric, exhibiting no observable ferroelectric phase transition at temperatures below liquid helium. However, it can transition into a ferroelectric state when adequately doped. Esswein and Spaldin link their computational findings to recently observed superconductivity on KTO surfaces, particularly highlighting its dependence on the surface orientation.

 The manuscript suggests that the electron-phonon interaction alone is insufficient to explain the surface-dependence observed in the superconducting transition temperature. This implies the presence of an unconventional mechanism, potentially mediated by polar soft modes.

 The article begins with a comprehensive summary of recent superconductivity measurements, effectively contextualizing the new computational results. While the methods used are adequately described, the manuscript primarily focuses on the physical implications of the results, making it accessible to a broad readership.

 One aspect that could benefit from further elaboration is the projection scheme used to relate bulk electron-phonon coupling to the surface. The authors employ the correspondence (001):Gamma-X; (011):Gamma-M; (111):Gamma-R, associating these high-symmetry lines with the wave-vector mediating a phonon pointing out of the respective surface area. However, it might be worth exploring the potential contribution of other phonon modes to the surface normal direction.

 Furthermore, the manuscript raises intriguing questions about the notably stronger electron-phonon direction along (001), according to Figure 3, despite the apparent suppression of superconductivity in this direction. This finding poses an interesting avenue for further investigation.

Is the study design appropriate and does the work have academic merit?

Yes

Is the work clearly and accurately presented and does it cite the current literature?

Yes

If applicable, is the statistical analysis and its interpretation appropriate?

Yes

Are all the source data underlying the results available to ensure full reproducibility?

Yes

Are the conclusions drawn adequately supported by the results?

Yes

Are sufficient details of methods and analysis provided to allow replication by others?

Yes

Reviewer Expertise:

Condensed matter and materials theory; ab initio materials modelling;

I confirm that I have read this submission and believe that I have an appropriate level of expertise to confirm that it is of an acceptable scientific standard.
